# Efficient Computational Design of a Scaffold for Cartilage Cell Regeneration

**DOI:** 10.3390/bioengineering5020033

**Published:** 2018-04-24

**Authors:** Tannaz Tajsoleiman, Mohammad Jafar Abdekhodaie, Krist V. Gernaey, Ulrich Krühne

**Affiliations:** 1 Department of Chemical and Biochemical Engineering, Technical University of Denmark, DK-2800 Kgs., Lyngby, Denmark; tantaj@kt.dtu.dk (T.T.); kvg@kt.dtu.dk (K.V.G.); 2 Department of Chemical and Petroleum Engineering, Sharif University of Technology, Tehran, Iran; abdmj@sharif.edu

**Keywords:** tissue engineering, CFD simulation, scaffold geometry optimization, micro-bioreactor operating conditions

## Abstract

Due to the sensitivity of mammalian cell cultures, understanding the influence of operating conditions during a tissue generation procedure is crucial. In this regard, a detailed study of scaffold based cell culture under a perfusion flow is presented with the aid of mathematical modelling and computational fluid dynamics (CFD). With respect to the complexity of the case study, this work focuses solely on the effect of nutrient and metabolite concentrations, and the possible influence of fluid-induced shear stress on a targeted cell (cartilage) culture. The simulation set up gives the possibility of predicting the cell culture behavior under various operating conditions and scaffold designs. Thereby, the exploitation of the predictive simulation into a newly developed stochastic routine provides the opportunity of exploring improved scaffold geometry designs. This approach was applied on a common type of fibrous structure in order to increase the process efficiencies compared with the regular used formats. The suggested topology supplies a larger effective surface for cell attachment compared to the reference design while the level of shear stress is kept at the positive range of effect. Moreover, significant improvement of mass transfer is predicted for the suggested topology.

## 1. Introduction

Cartilage diseases such as hyaline damages are among the most common skeletal health issues [1,2]. In case of advanced cartilage diseases, there is a considerable need for an external intervention due to the limited recovery ability of mature cartilage cells (chondrocyte), particularly for elderly patients. In an advanced case of tissue damage, when partial or complete cartilage transplantation is needed, tissue engineering demonstrates its potential as a candidate alternative for auto-graft/allograft tissue transplantation. In a successful tissue engineering procedure, physical and physiological properties of the target tissue are recreated into a new 3 dimensional cell structure during a complex cell culture process [3].

Optimized protocols and operating conditions can guarantee a successful tissue regeneration cell culture. The term operating condition is a general description, which covers the capacity of mass transfer as a function of cell culture strategy and physical environment characteristics influenced by the type of reactor. Mammalian cell cultures are generally operated under either static or dynamic cultivation principles. Mass transfer capacity of a static cell culture is mainly controlled by molecular diffusion within the growth environment [4]. Lack of nutrient supply and an increased chance of metabolite accumulation are the main bottlenecks in high-density static cell cultures. Using perfusion bioreactors is an alternative approach to increase mass transfer within a cell culture environment. In the case of mammalian cell cultures, shear stress can boost cell metabolism and proliferation rate [5,6,7,8]. This parameter is directly controlled by the induced flows in perfusion bioreactors. All these considerations illustrate the complexity of a dynamic cell culture and the necessity of having a comprehensive knowledge of the hydrodynamic environment of the cell culture.

**Figure 1:**
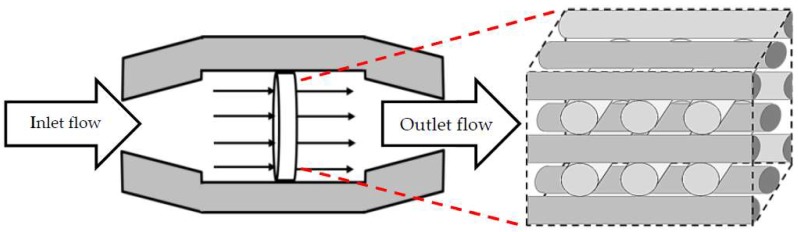
3D fibrous scaffold placed in a perfusion micro-bioreactor.

Present limitations of on-line monitoring techniques, particularly at the cell level, form one of the most persuading reasons to use mathematical modelling and simulation to estimate process variables. For instance, Williams et al. used computational fluid dynamics (CFD) simulation to quantify momentum and mass transfer within a cartilage cell culture in order to improve the design of a concentric cylinder-shape bioreactor [9]. In their work, they focused on flow field, shear stress profile and molecular oxygen distribution around a non-porous structure, represented as a three-dimensional (3D) cell growth space. Raimondi et al. used computational modelling to estimate fluid dynamic properties induced on a mature cartilage cell to characterize the effect of perfusion flow on the cell culture [10]. The coupled experimental model was extracted based on a dynamic cell culture on a biodegradable scaffold, which was exposed to a bi-directional flow of culture medium. Krühne et al. tried to directly use CFD to simulate the dynamic interactions between the liquid flow and growing cells in an exemplary pore, as a part of a porous scaffold. In this regard, they introduced a simple biological growth model based on Michaelis-Menten kinetics, combined with a shear stress term, to estimate the cell growth rate under fluid flow conditions [11]. Later, mathematical modelling and computational simulation were extensively used to study environmental conditions in various cell cultures [12,13]. Mathematical modelling and CFD simulations are not only used for characterizing a process behavior, but they are also helpful tools to optimize a process condition and improve the culture environmental design. This study presents a systematic approach to use computational predictions for more efficient 3D cell cultures. Hence, the first part of this paper focuses on mathematical modelling and CFD simulation of a 3D scaffold based cartilage cell culture within a perfusion bioreactor. In this part, a predictive model is introduced by considering various biochemical and physical factors. This model covers the main effective parameters such as nutrient supply, metabolite concentration and fluid shear stress on cartilage cells within a wide range of environmental conditions. The considered perfusion reactor and the investigated scaffold are shown in Figure 1. The developed model potentially helps to understand and characterize the cell culture behavior under perfusion flow conditions.

**Figure 2:**
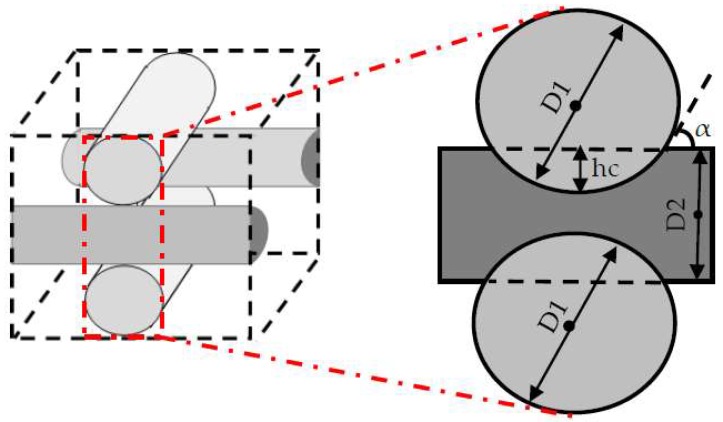
Scaffold design characteristics at the attachment point.

The studied scaffold is a common type of fibrous structure that is frequently reported for 3D cartilage regeneration [12,14,15,16]. This structure can be fabricated by assembling polymeric fibers with a repeated pattern, or by using 3D printers. Hossain et al. initially discussed the fluid velocity drop, downstream of the rear side of the fibers, as a design weakness of this structure. This fact is a considerable phenomenon, particularly in the case of relatively slow perfusion or low nutrient concentrations in the feed stream [17]. Hence, the effect of the scaffold design on the cell culture process needs to be investigated in more detail. Here, the scaffold design is represented by the fiber diameters and the compaction depth h_c_ at the attachment point between two fibers, which is formulated into the attachment angle α in Figure 2. The second part of the article focuses on using a CFD model to reach an improved scaffold design. Thereby, a new stochastic topology optimization algorithm is introduced to build a bridge between the characterization step and environmental design corrections and improvements. Accordingly, improved design dimensions for this class of scaffolds are proposed with respect to the fiber diameters and the attachment angles. In the last part of the paper, the effect of bioreactor operating conditions, such as perfusion velocity and medium nutrient concentration, on the culture efficiency is studied.

## 2. Mathematical Model

In order to predict a cartilage cell culture, a multi-phase model is developed by including three major domains, i.e., the solid fibrous scaffold, the perfusion medium and the dynamic biomass volume. The scaffold is assumed as a rigid impermeable structure, which provides cell attachment surfaces. This assumption simplifies the predictive model by eliminating mass transfer phenomena inside the solid phase. Furthermore, simulating the medium properties with the assumption of Newtonian behavior significantly reduces the complexity of the equations. In this model, the biomass domain represents a dynamic heterogeneous volume, attached to the solid surface, with various ratios of medium and cells volume fractions ($\varepsilon_{f}$ and $\varepsilon_{c}$, respectively). By changing the cell density during the cultivation, the ratio of volume fractions within the biomass domain changes as a function of time and location according to Equation (1). This equation is formulated assuming a constant solid phase volume.

(1) $$\varepsilon_{f}\;(x,\;y,\;z,\;t)+\varepsilon_{c}\;(x,\;y,\;z,\;t)=1,$$

The distributions of nutrients and metabolite concentrations in the biomass domain are mainly controlled by $\varepsilon_{c}$ and $\varepsilon_{f}$. The biochemical aspects of various nutrient concentrations in cartilage cell cultures were extensively investigated in previous studies [8,18,19]. For instance, oxygen and glucose are identified as the main nutrient supplies for mammalian cell metabolism. It is established that the oxygen uptake rate level in cartilage cell cultures is adjusted based on the glucose consumption rate [3,20,21]. Hence, glucose was selected as the main nutrient to be included into the predictive model. The distribution of glucose concentrations is calculated according to Equation (2).

(2) $$\frac{\partial }{\partial t}\left[\varepsilon_{f}\;\times c_{g}{}^{f}+\varepsilon_{c}\times c_{g}{}^{c}\right]=\nabla \times \left(D_{g}{}^{f}\times \nabla c_{g}{}^{f}+D_{g}{}^{c}\times \nabla c_{g}{}^{c}\right)-R_{g}-v\nabla \times c_{g}{}^{f},$$

Given Equation (2), $c_{g}{}^{f}$ and $c_{g}{}^{c}$ are the intrinsic average glucose concentrations, and $D_{g}{}^{f}$ and $D_{g}{}^{c}$ are diffusion tensors in the fluid (f) and the cell (c) respectively. It should be mentioned that intercellular mass transport is restricted to molecular diffusion as a function of cell physiology. The glucose consumption rate R_g_ is formulated according to Michaelis-Menten (M-M) kinetics in Equation (3), both as a function of cell density and intercellular glucose concentration:

(3) $$R_{g}=\frac{V_{m}\;\varepsilon_{b}\times \;c_{g}{}^{c}}{K_{m}+\varepsilon_{b}\times \;c_{g}{}^{c}},$$

where, $V_{m}$ and $K_{m}$ are the maximum specific glucose uptake rate and the half-saturation constant for glucose, respectively. The interfacial properties for the glucose concentration at the cell membrane were simplified by introducing the linear equilibrium coefficient $K_{eq}$ [22].

In this model, glucose breakdown resulting into lactate formation is considered as the main metabolic pathway for cartilage cells [20,21]. Governing equation of lactate concentration in fluid phase $c_{l}{}^{f}$ is shown in Equation (4).

(4) $$\frac{\partial }{\partial t}\left[c_{l}{}^{f}\right]=\nabla \times \left(D_{l}{}^{f}\times \nabla c_{l}{}^{f}\right)+R_{l}-v\nabla \times c_{l}{}^{f},$$

Rate of lactate formation $R_{l}$ is estimated to be two times of glucose consumption rate. The value of the lactate concentration is directly reflected by the pH level of the cell culture according to Equation (5) [23,24].

(5) $$PH=7.4-0.0406\;C_{l},$$

It should be considered that glucose has a double effect on cartilage cell culture. Lack of glucose essentially threatens the cell viability and growth rate, whereas an increase in glucose concentration proportionally boosts the cell metabolism rate and the correlated lactate production rate. Toxicity is the main outcome of a high metabolite concentration that is observed by dropping pH levels in an inefficient cell culture environment. Various cell types show different pH resistance [25] Cartilage cells are sensitive to an acidic environment with a pH value lower than 6.8. This condition is formulated as a step function S{pH} and the value of $\varepsilon_{c}$ is updated to $\varepsilon_{c}\times$ S{pH}. 

Mathematical modelling also provides the possibility of estimating the cell number and distribution. The cultured cell population is mainly controlled by three terms: proliferation, death and migration. The dynamic cell distribution during a cell culture can be quantified into $\varepsilon_{b}$ according to Equation (6) [26].

(6) $$\frac{\partial }{\partial t}\left[\varepsilon_{c}\times \rho_{c}{}^{b}\right]=\nabla \times \left(D_{c}\times \nabla \left(\varepsilon_{c}\times \rho_{c}{}^{b}\right)\right)+\left(r_{g}-r_{d}\right)\times \varepsilon_{c}\times \rho_{c}{}^{b}\;,$$

where, $r_{g}$ and $r_{d}$ are cell growth and death rate, respectively. $\rho_{c}{}^{b}$ is the intrinsic average cell mass density and $D_{c}$ is the cell effective diffusion tensor that indicates the migration term. The migration term is quantitatively negligible compared with the two other terms. Nutrients concentration, metabolite distribution and physical microenvironment properties (e.g., pH and fluid shear stress) are the main controlling parameters of the cell proliferation rate. In this study, the effect of metabolite concentration on cell culture is considered solely from an environmental (pH limiting range) point of view. The effect of the glucose concentration, cell density and local shear stress on the proliferation rate is given by Equation (7) [27].

(7) $$r_{g}=\;\frac{K_{g}\times c_{g}{}^{c}}{K_{c}\times \rho_{cell}\varepsilon_{c}+c_{g}{}^{c}},$$

where, $K_{c}$ and $\rho_{cell}$ are the saturation coefficient and cellular density respectively. In this equation, $K_{g}$ represents the effect of fluid shear stress on the cell proliferation rate. The stimulating influence of low range shear stress on the cell growth rate has been established in previous studies [3,12,13,27,28]. However, this effect changes to an inhibitory factor at a higher range of shear stress values, such that shear stress higher than 1 Pa has been reported as a damaging condition for the cell viability [12,13]. Equation (8) shows a modified correlation between the level of fluid induced shear stress and the cartilage cell growth rate.

(8) $$K_{g}=\{\begin{array}{cc} K_{g_{0}}(\alpha +\beta \tau ) & for\;\tau \in [0,0.1)Pa\\ K_{g_{0}}\times 11.326 & for\;\tau \in [0.1,0.6)Pa\\ K_{g_{0}}\times (2.5\times (1-\tau )\times 11.326) & for\;\tau \in [0.6,1)Pa\\ 0 & for\;\tau >1Pa \end{array},$$

In Equation (10), $K_{g_{0}}$ is the maximum growth rate under static culture conditions and α and β are constant parameters which are illustrated in Table 1. 

A transient scaffold based cartilage cell culture was simulated based on the above-mentioned model coupled with the Navier-Stokes and continuity equations [29]. The hydrodynamic behavior of the perfusion flow was predicted by applying a laminar model on a representative Newtonian fluid (water properties were considered for the description of the fluid phase). The boundary conditions for the fluid phase were set to a plug flow at the inlet interface, an opening outlet with relatively zero pressure and no-slip condition at the scaffold walls. The simulation was initialized by specifying the biomass domain as a uniform volume, with the viscosity of $\mu_{c}$ and the thickness of $d_{c}$ over the solid attachment surface. The local value of $\mu_{c}$ was set as a linear function of the cell volume fraction. By growing the cells population, the local value of $\mu_{c}$ increases proportionally with $\varepsilon_{c}$ within the range of 0.001 Pa.s, as the presence of no cells, to 1 Pa.s as a fully packed bulk of the cells. The calculated viscosity regulates the penetration rate of the fluid in the biomass domain. Moreover, the assumption of a monolayer cell culture was included by setting the value of $d_{c}$ to the dimension of one cartilage cell and limiting the upper bond of the cells distribution (cell/attachment area), according to the available attachment surface. The CFD simulation was performed using second order backward Euler approach and finally, the results were validated by available experimental data in the literature [30]. 

## 3. Methodology

**Figure 3:**
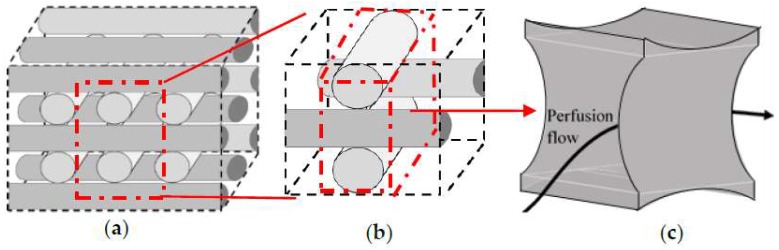
Geometry simplification (**a**) full structure, (**b**) conjunction of four fibers, (**c**) the studied simulation volume (unit).

As discussed in the introduction, the design of a scaffold and bioreactor operating conditions are critical parameters, which have to guarantee a balance between nutrient supply rate, metabolite removal performance and physical environmental conditions. Modelling and simulation are powerful tools for estimating the level of effective factors—such as nutrients concentrations and other environmental factors like shear stress—to predict the state of a cell culture under various operating conditions. One of the main advantages of process simulation is that it offers the opportunity to better understand the ongoing phenomena and diagnose the design weaknesses and the potential bottlenecks towards achieving an optimal design. In this overall perspective, the geometrical aspects of the scaffold design are investigated in order to systematically explore an improved design for more efficient cartilage cell cultures. Hence, a CFD based stochastic algorithm was developed according to the following steps:

Step 1: Division of the studied geometry into a network of repeated sections towards focusing only on a single unit due to the intensive computational demands. The selected unit has to include the main influence on the fluid dynamic conditions. Thus, the studied geometry is initially discretized into its structural repetition element (Figure 3b). Figure 3c shows a section of the fluid volume where the main hydrodynamic fluid phenomena are happening. This volume is labelled as the investigation ‘unit’ that is considered for detailed geometrical studies.

Step 2: Identification of an indicator parameter to quantitatively evaluate the overall cell culture conditions. In this case study, the dimensionless parameter ‘culture efficiency (CE)’ is introduced to evaluate the yield of the cell culture under various geometrical and operating conditions. The culture efficiency is defined in Equation (9).

(9) $$CE=\frac{{\left(\bar{cell\;density}\right)}_{final\;}-\;{\left(\bar{cell\;density}\right)}_{inital}}{{\left(\bar{cell\;density}\right)}_{initial}},$$

Step 3: Evaluation of various design possibilities with respect to the indicator parameter and fabrication feasibilities. The final state of the cell culture is mainly controlled by the mass transfer capacity into the culture environment and the level of hydrodynamically induced shear stress on the cells. On the other hand, for monolayer cell cultures such as for cartilage cells, the available amount of attachment surfaces for the growing biomass is a deterministic parameter [34,35]. Hence, the main challenge is to find an improved scaffold design which provides an efficient mass transfer over an extended attachment surface, while simultaneously keeping the level of shear stress at the supportive range of effect.

**Figure 4:**
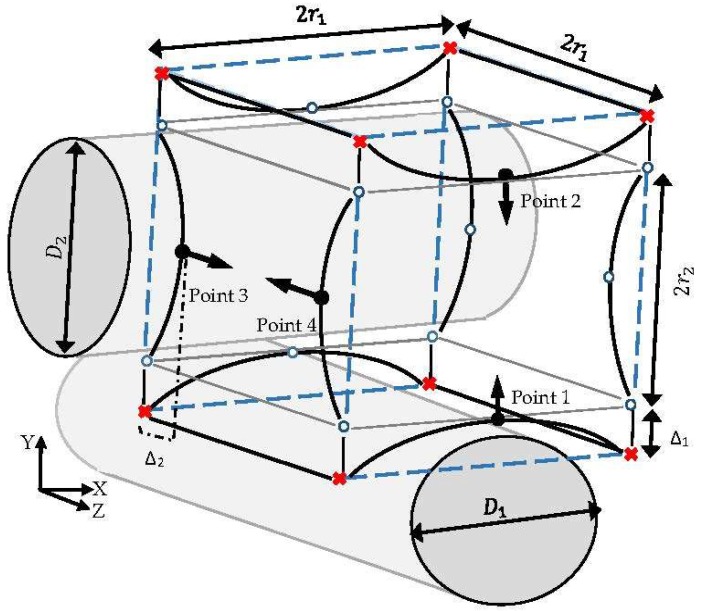
The studied unit: 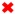 fixed point, 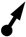 indicator point, 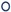 free point, 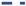 imaginary cube edges.

The estimation of the indicator parameter (CE) through the CFD simulation gives the opportunity of assessing the capabilities of various structures to fulfil a desired cultivation condition during a cell culture. Regarding the intensive computational efforts experienced when considering the complexity of the process for each scaffold candidate, the evaluation of all possible structures is not feasible. Therefore, a finite number of candidate structures are selected through a new stochastic method to reach an improved structure. Each candidate structure is specified by initially introducing an imaginary peripheral cube with dimension $2r_{1}$, which assigns the maximum possible volume for one unit. This cube is fixed by the allocation of 8 points at its corners, as shown in Figure 4. In this unit, the front and back fibers (fiber 3 and fiber 4) are named as the secondary fibers, which are positioned in relation to the top and bottom fiber diameters (fiber 1 and fiber 2; primary fibers) and the compaction depth_._ The diameters of the primary fibers are defined by introducing indicator points 1 and 2 at the center line of the cube’s front face in the Y axis direction. The normal distance Δ between each indicator parameter and the center of the closest imaginary cube’s edge illustrate the corresponding fiber’s diameter D according to Equation (10). Accordingly, secondary fibers are specified by indicating the coordinates of points 3 and 4 with respect to the closest imaginary edge at the cube’s left face. Movement of the indicator points from the cube’s edge in their corresponding faces (in the Y direction for points 1 and 2, and in the Z direction for points 3 and 4) indicates the main design characteristic parameters of various possible structures. The movements are specified by setting the displacement values Δ s. Moreover, 12 extra points, named ‘free point’, are used to illustrate the rest of the corners and edges in the simulation software. The locations of the free points are adjusted according to the coordinates of the indicator parameters in the three-dimensional space.

(10) $$D=\frac{r^{2}\;+\;\Delta^{2}}{\Delta },$$

**Figure 5:**
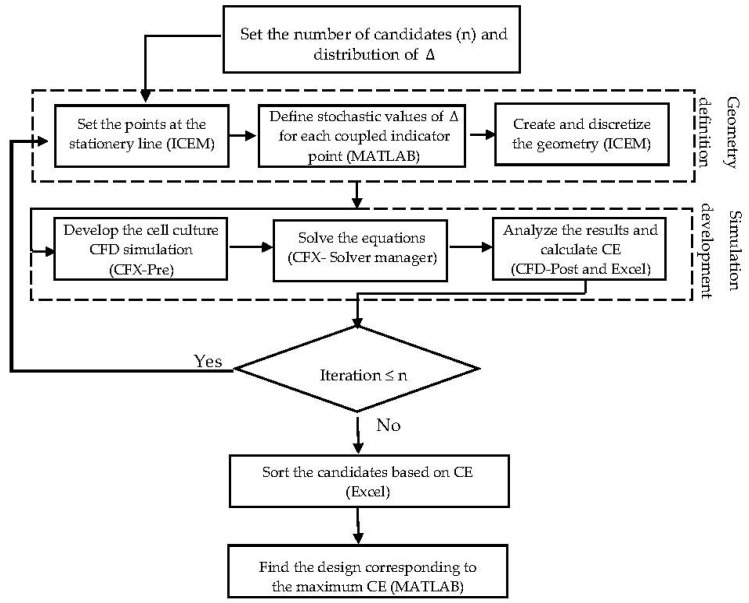
A schematic view of the stochastic semi-optimization routine.

To reach a feasible design, considering fabrication issues, it is assumed that the indicator points at the same face take similar displacement values Δ. The displacement value Δ is constrained by the maximum possible volume of one unit (volume of the imaginary cube). In this case study, the dimension of the imaginary cube was assumed to be 200 µm. Moreover, a random array of uniform distributed Δ was generated in the range of 0–100 μm, in order to specify and study a limited number of design candidates. The final proposed geometry is capable of showing better performance in terms of fulfilling the environmental requirements for the cell culture under fixed operating conditions. In this study, the explained procedures run sequentially under supervision of an interface developed in MATLAB^®^ R2014b (MathWorks, Natick, Massachusetts, MA, United States). Figure 5 shows the scheme of the developed interface. The presented algorithm automates the creation of a list of candidate designs based on a random distribution of Δ. The most efficient design among the studied structures is identified through a ‘generate and test’ strategy. The illustrated algorithm delivers a simple but efficient approach to directly benefit from the capability of the CFD simulation to improve the scaffold design. The interface provides an interactive communication platform between the CFD related software (ICEM CFD and ANSYS CFX^®^ 15.0) (Ansys Inc., Canonsburg, Pennsylvania, PA, USA) and the result processing software (Excel 2013). Regarding the numerical simulation requirements, the studied volumes were unstructurally discretized with the help of ‘Robust Octree’ meshing approach, with a global element seed size equal to 10. The codes are provided as a Supplementary Materials.

## 4. Results and Discussion

**Figure 6:**
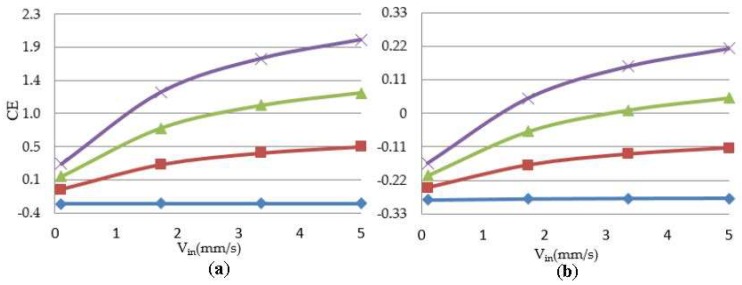
Correlation between cell culture efficiency vs. inlet perfusion velocity within the studied unit. $Gl_{in}$ = 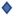 0.059 × 10^−3^ g/cm^3^, 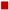 1.54 × 10^−3^ g/cm^3^, 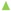 3.02 × 10^−3^ g/cm^3^, 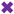 4.5 × 10^−3^ g/cm^3^ (**a**) initial cell density = 2 × 10^7^ cells/cm^3^; (**b**) initial cell density = 10 × 10^7^ cells/cm^3^.

As previously discussed, the velocity profile is a deterministic parameter in a cell culture under perfusion flow. The growing population of cells reflects the influence of the perfusion velocity during a culture. Figure 6 shows a representative simulation result of cartilage cell cultures for a range of inlet velocities and glucose concentrations within a sample scaffold unit.

The simulation results indicate the critical effect of fluid velocity on the cell culture efficiency, particularly in the case of high cell densities. Negative culture efficiency is interpreted as losing part of the cell population with time, under the specified operating conditions. The sensitivity of the cell culture to the inlet velocity profile is predicted to decrease at higher velocities. This behavior is explained by the multi-facial effect of shear stress on the cell growth rate (see Equation (8)). On the other hand, the sensitivity of the process to the flow velocity is a function of cell population. The populated cell culture shows higher resistance to the penetration of medium into the biomass volume, which overwhelms the intense influence of velocity on the cell culture.

**Figure 7:**
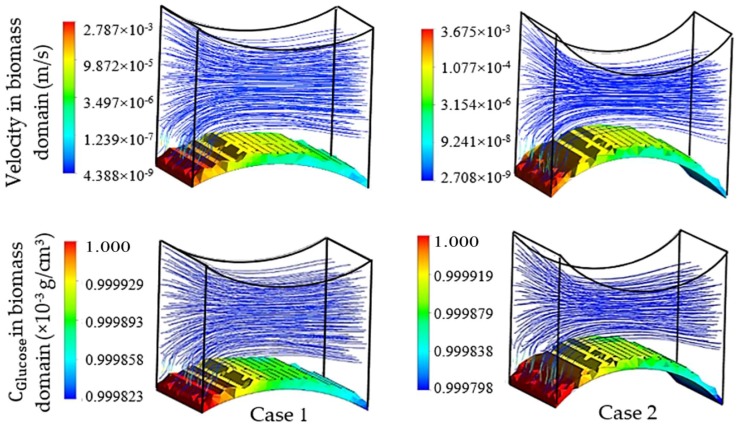
Physical effect of fiber 1 on the cell culture with initial cell density = 5 × 10^7^ cells/cm^3^ in the biomass phase, inlet velocity = 3 mm/s and inlet glucose concentration = 1 × 10^−3^ g/cm^3^, $D_{1}^{case1}=520\;\mu m$, $D_{2}^{case1}=0\;\mu m$ and $D_{1}^{case2}=250\;\mu m$, $D_{2}^{case2}=0\;\mu m$.

The velocity profile in the specified unit is also a strong function of the scaffold design. In order to individually investigate the geometric effect of each pair of fibers on the fluid dynamics of the cell culture environment, two sets of simulation settings were considered. Table 2 gives an overview of the simulated configurations and the results:

The first group of simulations was initialized by the presence of a homogeneous biomass domain on the surface of the bottom fiber with an average thickness of one cartilage cell. In order to study the individual effect of each pair of fibers on the cell culture, the left-right pair (non-cultured fibers) was initially replaced by flat walls with setting Δ_3_ and Δ_4_ to 0. Transient simulation results of cartilage cell cultures after 10 h real time are shown in Figure 7. In this figure, the influence of the cultured fiber characteristics on the velocity profile and glucose distribution in biomass domain is shown for two different designs. The reported CFD results in this section were achieved by choosing the ‘Second Order Backward Euler’ solver with a constant time step (1 h), which is below the time dependency threshold of the simulation.

As mentioned previously, a reduction in mass transfer and nutrient supply is a direct consequence of the velocity drop at the rear side of the fibers. Although, the difference between the maximum and minimum concentrations in both designs of the unit was estimated to be small, this difference can potentially reach a significant value when the complete scaffold is considered. The curvature corresponding to the present part of the fiber in a unit is named as the effective curvature. The effective curvature is determined by a given fiber’s diameter, the attachment angle and the size of the imaginary cube (Figure 5). The simulation results indicate the influence of the effective curvature on the fluid profile. As Figure 7 shows, an increase in the level of effective curvature from case 1 to case 2 results in a higher velocity drop in the downstream. An intense velocity gradient in perfused medium is directly reflected in the mass transfer rate profile, which consequently increases the possibility of ending with a significant level of heterogeneity in the biomass distribution. This result indicates a challenge in achieving a homogeneous cell distribution within a regenerated tissue. However, the heterogeneity issue can be seen as a potential in another perspective, such as for controlling cell density distribution within a scaffold by using various perfusion velocity profiles. Histology studies of natural cartilage tissue indicated the presence of distinctive zones with respect to cell distribution and morphology [33]. Usage of various scaffold designs gives the possibility of controlling the cell distribution within an engineered tissue in order to mimic the natural tissue [16].

The specified curvature also has a direct influence on the level of induced shear stress by the flow. Simulation results predict a relatively higher maximum shear stress in biomass domain for higher curvature (0.16 Pa in case 1 and 0.4 Pa in case 2), while the average parameter indicates a lower value in the same design (average shear stress in biomass phase = 0.013 Pa in case 1 and 0.011 Pa in case 2). Since for a cell culture the average value of shear stress has a higher level of importance compared to its local maximum value, a lower curvature of the cultured surface is predicted to be more desirable. On the other hand, the available surface area for the cell attachment is a critical factor in the design of an efficient cell culture structure [34,35]. The cell attachment surface area (A) in the specified unit is a direct function of the effective curvature. Hence, a larger surface area is calculated for higher curvature in case 2 (A = 4.026 × 10^−4^ cm^2^ for case 1 and A = 4.93 × 10^−4^ cm^2^ for case 2). Accordingly, a higher cultured curvature can provide more surface area for the cell attachment.

**Figure 8:**
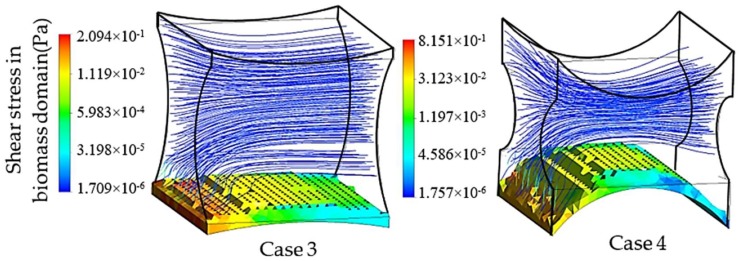
Hydrodynamic effect of the fiber location on the cell culture with initial cell density = 5 × 10^7^ cells/cm^3^ in the biomass phase, inlet velocity = 3 mm/s and inlet glucose concentration = 4.5 × 10^−3^ g/cm^3^, $D_{1}^{case3}=520\;\mu m$, $D_{2}^{case3}=340\;\mu m$ and $D_{1}^{case4}=250\;\mu m$, $D_{2}^{case4}=145\;\mu m$.

Similarly, the second group of simulations was initialized in the presence of a biomass domain on the surface of the bottom fiber while including the other two fibers in the simulation unit. Accordingly, two different design characteristics for the studied unit are shown in Figure 8. These simulations aim at studying the effect of the second pair of fibers as non-cultured fibers on the velocity and shear stress profile. The induced fibers mainly have an effect on the fluid dynamics of the perfusion flow by adjusting the cross-sectional area. Smaller cross-sectional areas regarding the position and characteristics of four fibers result in a higher perfusion velocity and shear stress in the unit. Transient simulation of a cartilage cell culture after 10 h real time indicates 0.08 Pa average shear stress in case 3 with 2.357 × 10^−8^ m^2^ minimum cross-sectional area compared to 0.18 Pa in case 4, with a 1.240 × 10^−8^ m^2^ minimum cross-sectional area. A decrease in the cross-sectional area is a beneficial factor to increase the shear stress within the positive range of effect. However, simultaneously the chance of blocking the unit increases during the cultivation.

**Figure 9:**
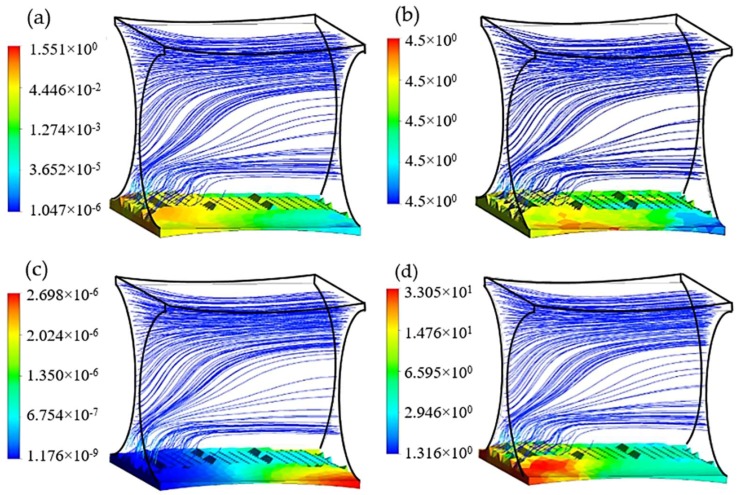
Cartilage cell culture within the improved geometry after 30 days cultivation with initial cell density = 5 × 10^7^ cells/cm^3^ (**a**) Shear stress (Pa); (**b**) Glucose concentration (kg/m^3^); (**c**) Lactate concentration (kg/m^3^) and (**d**) Cell density × 10^7^ (cells/cm^3^) in biomass domain.

The efficiency of the presented method to design an improved fibrous scaffold was investigated by assuming that the biomass domain is only present on the surface of one fiber in the studied unit. This assumption gives a better understanding of the scaffold geometric effect on cell culture efficiency while the potential of the routine in a specified case study is evaluated. Using this assumption gives the possibility of dip investigation on the effect of not only cultured surfaces, but also the possible nonculture ones on controlling the cell culture environment. The presence of nonculture surfaces could potentially be considered, for instance, to mimic the multi-zonal morphology of a tissue. According to the common design of the scaffold, the initial diameter of all 4 fibers was set to 200 µm. After studding 300 design candidates, the improved geometry was obtained with displacement values of |ΔY| = 7 µm for points 1 and 2, and |ΔZ|= 90 µm for points 3 and 4, which are equivalent to the improved dimensions $D_{1}$ = 1435 µm and $D_{2}$ = 182 µm for fiber 1 and 2 respectively. These values also indicate an attachment angle of $\sim 0^{{}^{\circ}}$ between the two fibers. A transient simulation of the cartilage cell culture for 3 mm/s inlet fluid velocity and 4.5 × 10^−3^ g/cm^3^ glucose concentration within the improved structure is shown in Figure 9. The simulation is initialized by considering a monolayer biomass domain attached to the bottom fiber with constant cell density 5 × 10^7^ cell/cm^3^.

Among the studied candidates, this geometry delivers the best performance in terms of nutrient supply balanced with metabolic growth rate, available attachment surface (A = 4.01 × 10^−8^ m^2^), and flow induced average shear stress ($\;\bar{\tau }$ = 0.17 Pa). The relatively large radius of the cultured fiber guarantees an adequate level of effective curvature for the passing flow to keep the capacity of mass transport approximately constant throughout the structure. On the other hand, the second pair of fibers has the main influence on adjusting the level of shear stress on the cell culture by optimizing the cross-sectional area for perfusion flow. In this case study, the final average cell population was predicted to reach 11.68 × 10^7^ cells/cm^3^ after 30 days cultivation, which shows up to 24% improvement compared to the reference design (*D*_1_ = *D*_2_ = 200 µm).

**Figure 10:**
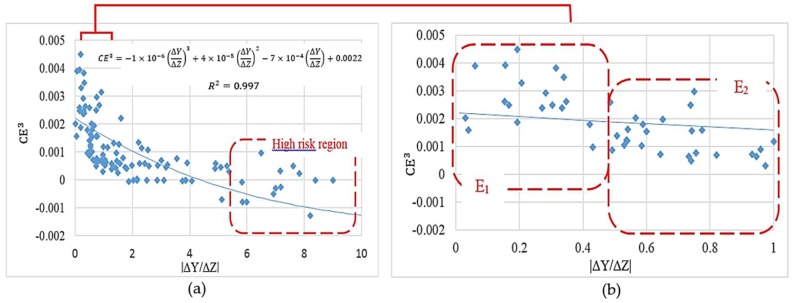
The effect of scaffold design on the cartilage cell culture efficiency, (**a**) full simulation results; (**b**) simulation results in a range of |ΔY/ΔZ| ∈ [0–1].

This method was also applied to a more realistic case study in which the attachment of the biomass phase over the entire fiber surfaces was considered. After studding 200 design candidates, the improved characteristic parameters were achieved accordingly: $D_{1}$ = 605 µm, $D_{2}$ = 204 µm and α = 20°. The improved dimensions are expressed by displacement values |ΔY| = 17 µm for point 1 and 2, and |ΔZ| = 40 µm for point 3 and 4. This scaffold can be assembled by various fabrication methods and new outstanding technologies such as advanced 3D-printing techniques. The simulation results of 200 design candidates are shown in Figure 10a. The collective results indicate an inverse correlation between the fiber’s diameters ratio D_1_/D_2_ (proportional to the corresponding value ΔY/ΔZ) and the cell culture efficiency.

The simulation results in Figure 10b show a considerable risk of negative culture efficiency for |ΔY/ΔZ| > 5.5. Moreover, designs in the range of |ΔY/ΔZ| ∈ [0–1] are expected to deliver the highest cell culture efficiencies within the studied range. This range is classified into two major regions $E_{1}$ and $E_{2}$ in Figure 10b. Although the design criterion $\left|\frac{\Delta Y}{\Delta Z}\right|\in E_{1}$ was predicted to deliver a more appropriate culture environment, the design criterion $\left|\frac{\Delta Y}{\Delta Z}\right|\in E_{2}$ proposes more feasible designs when considering the ease of fabrication.

## 5. Conclusions

Nutrient supply, metabolite concentrations and flow-induced hydrodynamic conditions are the main controlling parameters for cartilage cell culture in a perfusion bioreactor [3,13,20]. Using mathematical modelling and CFD simulation helps to predict the state of the cell culture during the process with respect to effective parameters such as perfusion flow rate, geometric design, influent glucose concentration etc.

In the present study, the potential of modelling and CFD simulation to design more efficient cell culture processes was investigated. Hence, a complex multiphase model, including the fluid flow and the growing cells, was presented to predict the state of the culture during a cartilage cell culture under a perfusion flow. In this model, the effects of glucose concentration, shear stress and pH of the environment on the cells were included. It should be considered that this model does not cover the effect of other involved nutrients such as oxygen on the cell metabolism, which requires further investigation. However, the developed model and the corresponding CFD simulation provide valuable information about the requirements, challenges and considerations for an efficient cell culture from a scaffold design point of view. In order to take advantage of this capability, a stochastic algorithm was introduced to evaluate various structure candidates, and to select the most efficient one. Based on that, an automated routine was developed in order to study various geometries virtually, within a simulation software environment, and to assess new, potentially useful structures for the cell culture. Here, a frequently used fibrous scaffold was implemented as a case-study. The proposed improved design has demonstrated to deliver significantly higher efficiency compared with the reference design in terms of mass transfer rate and hydrodynamic parameters. As the result, up to 24% improvement in the final cell population was predicted for the new design.

This promising approach can be considered for other types of scaffolds by numerically evaluating different geometrical structures within a relatively shorter operation time, while the experimental effort is mostly limited to the validation of the model.

## Figures and Tables

**Table 1:** Numerical values of the model parameters used in the simulations.

Definition	Value	Reference
Cell density, $\rho_{cell}$	0.182 [g/cm^3^]	[13]
Glucose diffusion tensor in fluid, $D_{g}{}^{f}$	1 × 10^−5^ [cm^2^/s]	[31]
Glucose diffusion tensor in the cell, $D_{g}{}^{c}$	1 × 10^−6^ [cm^2^/s]	[31]
Glucose diffusion tensor in the cell, $D_{l}{}^{f}$	1.4 × 10^−5^ [cm^2^/s]	[23]
Glucose maximal consumption rate, $V_{m}$	3.9 × 10^−5^ [kg/m^3^s]	[32]
Glucose half saturation constant, $K_{m}$	6.3 × 10^−3^ [kg/m^3^]	[32]
$K_{g_{0}}$	5.8 × 10^−6^ [1/s]	[13]
Equilibrium coefficient, K_eq_	0.1	[26]
α	0.8761	[13]
β	0.1045 × 10^3^ [1/Pa]	[13]
$d_{c}$	13 [μm]	[33]

**Table 2:** Overview of the simulation exploratory workspace for an improved scaffold design.

Figure	Case	Case Configuration	Diameters	Result
3	Reference 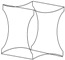	Biomass present on surface of all four fibers	*D*_1_ = *D*_2_ = 200 μm	
7	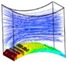 Case 1 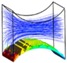 Case 2	Biomass present only on surface of one fiber from the pair 1. Presence of 1 pair of fibers in the unit	The cultured fiber: $D_{1}^{case1}=520\;\mu m$ $D_{1}^{case2}=250\;\mu m$ Non_cultured pair $D_{2}=0\;\mu m$	$EC_{case2}$ > $EC_{case1}\;$* A higher velocity drop at the rear side of the cultured fiber in case 2 A higher maximum shear stress and lower average shear stress in case 2 A higher cell attachment area in case 2
8	Case 3 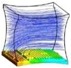 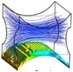 Case 4	Biomass present only on surface of one fiber from the pair 1. Presence of 2 pairs of fibers in the unit	The cultured fiber: $D_{1}^{case3}=520\;\mu m$ $D_{1}^{case4}=250\;\mu m$ $D_{1}\neq 0\;\mu m$ Non_cultured pair: $D_{2}^{Case\;3}$ > $D_{2}^{Case\;4}$ $D_{2}\neq 0\;\mu m$	$EC_{case4}$ > $EC_{case3}\;$* A higher velocity drop at the rear side of the cultured fiber in case 4 A higher cell attachment area in case 2 $A_{3}>A_{4}\;$** A lower flow cross-sectionional area in case 4 A higher level of shear stress in case 4
9	Improved design 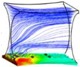	Biomass present only on surface of one fiber from the pair 1. Presence of 2 pairs of fibers in the unit	The cultured fiber $D_{1}=1435\;\mu m$ Non_cultured pair: $D_{2}=185\;\mu m$	24% improvement is predicted compared with the reference design

* EC: Effective curvature of the cultured surface; ** A: minimum intersection area for the perfusion flow.

## Supplementary Materials

The following are available online at http://www.mdpi.com/2306-5354/5/2/33/s1.

## Nomenclature

A	surface area [m^2^]
$c_{g}{}^{f}$	average glucose concentration in fluid [kg/m^3^]
$c_{g}{}^{c}$	average glucose concentration in cell [kg/m^3^]
$C_{l}$	lactate concentration [mol/m^3^]
D	fiber’s diameter [m]
$d_{c}$	thickness of the biomass domain [mm]
$D_{g}{}^{f}$	diffusion coefficient of glucose in fluid [cm^2^/s]
$D_{g}{}^{c}$	diffusion coefficient of glucose in cell [cm^2^/s]
$K_{c}$	saturation coefficient
$K_{g_{0}}$	maximum growth rate under static culture conditions [1/s]
$r_{g}$	cell growth rate [1/s]
$r_{d}$	cell death rate [1/s]
$R_{g}$	Glucose consumption rate [kg/m^3^ s]
$V_{m}$	maximum glucose consumption rate [kg/m^3^ s]
$K_{m}$	half-saturation constant for glucose [kg/m^3^]
$\varepsilon_{f}$	fluid volume fraction
$\varepsilon_{c}$	cell volume fraction
$\rho_{c}{}^{b}$	average biomass density [g/cm^3^]
$\rho_{cell}$	cellular density [g/cm^3^]
τ	shear stress [Pa]
Δ	moving point displacement value [µm]
α	attachment angle [Rad]
h_c_	compaction depth [µm]
$\mu_{c}$	local viscosity of the biomass domain [Pa. s]
