# Supplementary material for: Efficient Computational Design of a Scaffold for Cartilage Cell Regeneration
Source: Bioengineering (Basel). 2018 Apr 24;5(2):33. doi: 10.3390/bioengineering5020033 (PMC6027378; doi:10.3390/bioengineering5020033)
Supplement: Supplementary file 1 [file bioengineering-05-00033-s001.pdf]

Efficient computational design of a scaffold for cartilage cell regeneration (Supplementary document)

Tannaz Tajsoleiman [1], Mohammad J. Abdekhodaie [2] Krist V. Gernaey [1] and Ulrich Krühne [1,*]

This document contains the implemented MATLAB code to developed the discussed interface between MATLAB ® R2014b, ICEM CFD, and ANSYS CFX ® 15.0 and Excel 2013.

- The red sentences include some guides for better understanding the codes
- The red sentences needs to be revised for new case studies

- **Part 1**

```
clear all
clc
 cell0=7;  %initial cell density is equal to 7×107 cells/cm3
 gl0=4.5;  %inlet glucose concentration is equal to 4.5×10-3 g/cm3
 v0=2;     %inlet flow velocity is equal to 2mm/s


%% Set the points at the stationery line (ICEM)

 x2=100;
 y2=0;
 z4=0;
 y4=100;
 x7=100;
 y7=200;
 z12=200;
 y12=100;
 y20=0;
 y19=200;
  randy1=0;
  randx=0;
  randy2=0;
  randz=0;
  xx22=x2+randx;
  xx77=x7-randx;
  yy22=y2+randy1;
  yy77=y7-randy1;
  yy2020=yy22+1;
  yy1919=yy77-1;
% % %
  zz44=z4+randz;
```

```
    zz1212=z12-randz;
    yy44=y4+randy2;
    yy1212=y12-randy2;
% % %
   x22=mat2str(xx22);
   x77=mat2str(xx77);
   y22=mat2str(yy22);
   y77=mat2str(yy77);
   y2020=mat2str(yy2020);
   y1919=mat2str(yy1919);
% % % %
   z44=mat2str(zz44);
   z1212=mat2str(zz1212);
   y44=mat2str(yy44);
   y1212=mat2str(yy1212);

n=300;  %The number of candidate structures
num=1;
for i=1:n
    result(num,1)=randx;
    result(num,2)=randy1;
    result(num,3)=randz;
    result(num,4)=randy2;
    [f_yield,f_density]=model    (cell0,gl0,v0,x22,y22,z44,y44,z1212,y1212,x77,y77,y2020,y1919,num,randy1)

    filename=('name and directory');
    sheet='sheet name';
    xlRange='A?'
    density0=xlsread(filename,sheet,xlRange)
    result(num,5)= (f_density-density0)/density0;
    result(num,6)= density0;
    result(num,7)= f_density;


    %random delta1 and delta2 generation

    randy1=randi([0 90]);
    RR=((200-2*randy1)/2)-2;
    if RR>0
      R=min([RR,90]);
      randz=randi([0 R]);
    else
      randz==0
    end

    randx=0
    randy2=0
```

```
    %% Set the points at the stationery line (ICEM)

        xx22=x2+randx;
        xx77=x7-randx;
        yy22=y2+randy1;
        yy77=y7-randy1;

        zz44=z4+randz;
        zz1212=z12-randz;
        yy44=y4+randy2;
        yy1212=y12-randy2;
          if yy22+2>0
          yy2020=yy22+2;
          else
            yy2020=2;
          end
          if yy77-2<200
          yy1919=yy77-2;
          else
            yy1919=198;
          end

          x22=mat2str(xx22);
          x77=mat2str(xx77);
          y22=mat2str(yy22);
          y77=mat2str(yy77);

          z44=mat2str(zz44);
          z1212=mat2str(zz1212);
          y44=mat2str(yy44);
          y1212=mat2str(yy1212);
          y2020=mat2str(yy2020);
          y1919=mat2str(yy1919);

          num1=num+1;
          num=num1;
end


%find the improved design
for i=1:n
   opt_result(i,1)=result(i,5);
end
M=max(opt_result)


%%regenerate the chosen design
```

```
filename = 'resultdata.xlsx';
sheet = ‘sheetname’;
xlRangea = 'A?';
xlRangeb = 'A?';
title={'randx','randy1','randz','randy2','f_yield','inital density','f_density*1e13'};
A=result;
xlswrite(filename,A,sheet,xlRangea)
xlswrite(filename,title,sheet,xlRangeb)
index=find(opt_result(i,1)==M)
opt_geo=result(index,:);
randy1=opt_geo(1,2);
randx=opt_geo(1,1);
randz=opt_geo(1,3);
randy2=opt_geo(1,4);

 xx22=x2+randx;
 xx77=x7-randx;
 yy22=y2+randy1;
 yy77=y7-randy1;

 zz44=z4+randz;
 zz1212=z12-randz;
 yy44=y4+randy2;
 yy1212=y12-randy2;
 if yy22+2>0
 yy2020=yy22+2;
 else
   yy2020=2;
 end
 if yy77-2<200
 yy1919=yy77-2;
 else
   yy1919=198;
 end

x22=mat2str(xx22);
x77=mat2str(xx77);
y22=mat2str(yy22);
y77=mat2str(yy77);

z44=mat2str(zz44);
z1212=mat2str(zz1212);
y44=mat2str(yy44);
y1212=mat2str(yy1212);
y2020=mat2str(yy2020);
y1919=mat2str(yy1919);
```

- **Part 2**

```
function [out1,out2]=model (cell0,gl0,v0,x22,y22,z44,y44,z1212,y1212,x77,y77,y2020,y1919,num,randy1)
dis_concentration=distribution2(0.0002,0.0002,cell0,randy1);
dis_glucose0=mat2str(gl0);
dis_velocity0=mat2str(v0);
dis_cell0=mat2str(cell0);

% Opens the .pre file and format it into cell arrays
    Z = fopen('project2.pre');
    B = textscan(Z,'%s %s %s %s %s %s %s %s %s %s %s %s %s %s %s %s %s %s %s %s %s %s %s %s');
    C =
horzcat(B{1,1},B{1,2},B{1,3},B{1,4},B{1,5},B{1,6},B{1,7},B{1,8},B{1,9},B{1,10},B{1,11},B{1,12},B{1,13},B{1,14},B{1,
15},B{1,16},B{1,17},B{1,18},B{1,19},B{1,20},B{1,21},B{1,22},B{1,23},B{1,24});

% Insertion of the numbers into the file *.pre
   C{19,3}=(dis_concentration); %Data
   C{29,3}=(dis_glucose0); %glucose0
   C{141,4}=(dis_velocity0); %velocity0
   C{64,3}=(dis_cell0); %cell0

% Convert the matrix into a file *.pre
[nrows,ncols]= size(C);
filename='pro.pre';
fid=fopen(filename,'W');
for row=1:nrows;
    fprintf(fid,'%s %s %s %s %s %s %s %s %s %s %s %s %s %s %s %s %s %s %s %s  %s %s %s
        %s\r\n',C{row,:});
end
fclose all;
delete ('project2.pre');

!ren pro.pre project2.pre

% Opens the .rpl file and restructure it into cell arrays
    Z = fopen('project.rpl');
    B = textscan(Z,'%s %s %s %s %s %s %s %s %s %s %s %s %s %s %s %s %s %s %s %s %s %s %s %s');
    C =
horzcat(B{1,1},B{1,2},B{1,3},B{1,4},B{1,5},B{1,6},B{1,7},B{1,8},B{1,9},B{1,10},B{1,11},B{1,12},B{1,13},B{1,14},B{1,
15},B{1,16},B{1,17},B{1,18},B{1,19},B{1,20},B{1,21},B{1,22},B{1,23},B{1,24});

C{2,3}=(x22);
C{3,3}=(y22);
C{4,3}=(z44);
C{5,3}=(y44);
C{6,3}=(x77);
```

```
C{7,3}=(y77);
C{8,3}=(z1212);
C{9,3}=(y1212);
C{10,3}=(y2020);
C{11,3}=(y1919);

 % Convert the matrix into a file *.rpl
[nrows,ncols]= size(C);
filename='pro.rpl';
fid=fopen(filename,'W');
for row=1:nrows;
        fprintf(fid,'%s %s %s %s %s %s %s %s %s %s %s %s %s %s %s %s %s %s %s %s %s %s %s
        %s\r\n',C{row,:});
end
fclose all;
delete ('project.rpl');
!ren pro.rpl project.rpl

%run the simulation
! "C:\directory\icemcfd.bat" -batch -script "C:\directory\project.rpl"
! "C:\directory\cfx5pre.exe" -batch "C:\directory\project2.pre"
! "C:\directory\cfx5solve.exe" -def "C:\directory\project.def" -start-method "Platform MPI Local Parallel"
-part 4 -pri 1 -double -batch -chdir "C:\directory"
! "C:\directory\cfdpost.exe" -batch "C:\directory\project2.cse" "C:\directory\project_001.res"
out1=disyield
out2=discell

end
```

- **part 3:**
  - To prepare and run the simulation through MATLAB, three supplementary files are needed: 1. a '.rpl' file which includes the ICEM scripts to create geometry and generate the mesh; 2. a '.pre' file which includes the CFX scripts to set the simulation; and 3. a '.cse' file which includes the CFD post scripts to analysis the CFD simulation and extracts the CE values.
